# Autoimmune Hemolytic Anemia in a Patient With COVID-19 Pneumonia: A Case Report on a Rare Presentation

**DOI:** 10.7759/cureus.49067

**Published:** 2023-11-19

**Authors:** Umair Khizer, Jonathan Scott, Akshit Chitkara, Shukaib Arslan, Sonia Shoukat

**Affiliations:** 1 Internal Medicine, University of California, Riverside School of Medicine, Riverside, USA; 2 Hematology/Hematopoietic Cell Transplant, City of Hope National Medical Center, Duarte, USA

**Keywords:** autoimmune hemolytic anemia treatment, steroids in hemolytic anemia, autoimmune hemolytic anemia (aiha), covid-19 pneumonia, autoimmune hemolytic anemia covid19

## Abstract

Patients with severe acute respiratory syndrome coronavirus 2 (SARS-CoV-2)/coronavirus disease 2019 (COVID-19) pneumonia can have a range of clinical presentations ranging from being asymptomatic to having severe acute respiratory syndrome (SARS). Autoimmune hemolytic anemia (AIHA) is a very rare presentation of COVID-19.

We present the case of a 67-year-old male with a past medical history of chronic obstructive pulmonary disease (COPD) who presented to the emergency department (ED) with shortness of breath and was found to be COVID-19-positive. His laboratory results demonstrated autoimmune hemolytic anemia with decreased hemoglobin (Hgb), elevated lactate dehydrogenase (LDH), decreased haptoglobin, peripheral smear showing spherocytes, and a positive direct antiglobulin (Coombs) test. The patient was started on glucocorticoids, but his hemoglobin continued to worsen. The dose of glucocorticoids was increased significantly, and his hemoglobin started improving with the resolution of hemolysis.

Autoimmune hemolytic anemia is usually treated with glucocorticoids, but escalating glucocorticoid doses increases the risk of side effects. This case report highlights the importance of further research needed to establish guidelines for AIHA in the context of COVID-19 pneumonia.

## Introduction

An enveloped positive-stranded ribonucleic acid (RNA) virus causes coronavirus disease 2019 (COVID-19) [1]. Symptoms can range from asymptomatic to severe acute respiratory syndrome (SARS) with a multitude of complications. These complications can range from secondary infections, Guillain-Barré syndrome, pulmonary embolism, arrhythmias, cardiogenic shock, acute stroke, immune thrombocytopenia, and hemolytic anemia [1]. However, autoimmune hemolytic anemia (AIHA) in the setting of COVID-19 has rarely been reported in the literature [2-6]. In this report, we present one such case where a 67-year-old male was admitted for symptomatic COVID-19 with hypoxia and was found to have AIHA. It was treated with escalating doses of dexamethasone rather than the recommended 6 mg/day for symptomatic COVID-19 infection, which led to the resolution of anemia and increased hemoglobin.

## Case presentation

A 67-year-old male with a past medical history of chronic obstructive pulmonary disease (COPD) on 2 L home oxygen, hypertension, and benign prostatic hyperplasia presented to the emergency room with chest pain that started one week prior to presentation. The chest pain was retrosternal, non-radiating, pressure-like, 7/10, gradual in onset but increased gradually, relieved with rest, and associated with shortness of breath. Vitals showed 88% oxygen saturation on a 2 L nasal cannula that improved to 97% on a 4 L nasal cannula, blood pressure of 103/57 mmHg, respiratory rate of 20/minute, heart rate of 90/minute, and temperature of 37°C. The laboratory values are shown in Table 1.

**Table 1:** Laboratory values WBC: white blood cell, Hgb: hemoglobin, FEU: fibrinogen equivalent units, LDH: lactate dehydrogenase

Laboratory value	Result	Normal range	Abnormal/normal
WBC count	12.7 cells/mcL	4,000-11,000 cells/mcL	Abnormal
Hgb	7.1 g/dL	12-16 g/dL	Abnormal
Platelets	200,000 cells/mcL	150,000-450,000 cells/mcL	Normal
D-dimer	3.18 mcg/mL	Up to 0.5 mcg/mL (FEU)	Abnormal
Total bilirubin	4.2 mg/dL	0.3-1.2 mg/dL	Abnormal
LDH	665 U/L	140-280 U/L	Abnormal
Haptoglobin	<10 mg/dL	30-200 mg/dL	Abnormal
Ferritin	953 ng/mL	30-400 ng/mL	Abnormal
Procalcitonin	0.25 ng/mL	Up to 0.05 ng/mL	Abnormal
Troponin	0.058 ng/mL	Up to 0.04 ng/mL	Abnormal

**Figure 1:**
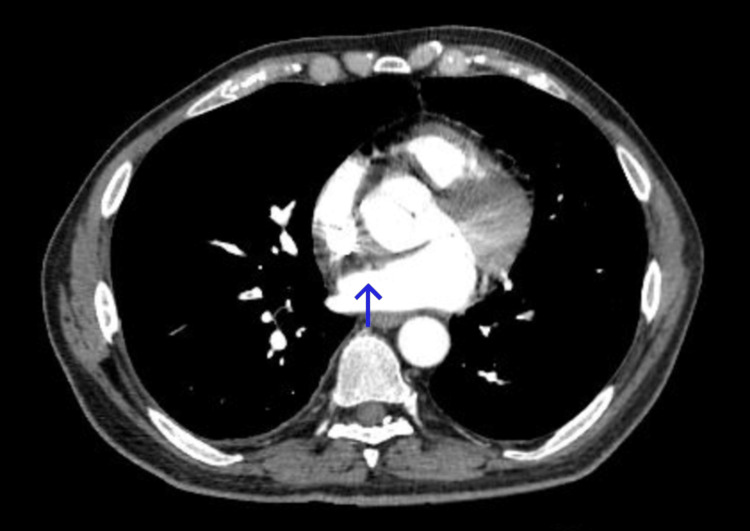
Computed tomography angiography of the chest showing a filling defect in the right lower lobe branch (arrow)

COVID-19 polymerase chain reaction (PCR) nasal swab test was positive. There was no evidence of melena, bleeding, hematoma, petechiae, or bruising in history or on physical examination. His peripheral smear showed moderate anisocytosis, polychromasia, macrocytosis, and spherocytes. The patient’s computed tomography pulmonary angiography revealed a small burden of pulmonary emboli (PE) in the peripheral pulmonary arteries bilaterally (Figure 1) showing a filling defect in the right lower lobe branch. His lower extremity ultrasound revealed no evidence of deep vein thrombosis bilaterally. An electrocardiogram (EKG) showed an old right bundle branch block. Physical examination showed ecchymosis on the right upper thigh but was otherwise normal.

The patient was admitted for acute hypoxemic respiratory failure secondary to COVID-19 pneumonia. The patient’s troponin trended upward and peaked at 0.42 ug/mL. The patient was started on a heparin infusion for pulmonary emboli with hypoxemia with SpO2 below 92%. The troponin elevation was deemed to be from mismatched myocardial oxygen supply and demand that was not related to unstable coronary artery disease (type 2 non-ST elevation myocardial infarction). The bedside echocardiogram did not show right heart strain. We consulted cardiology for elevated troponin and hematology for the anemia. The patient was started on dexamethasone 6 mg intravenous (IV) once daily for COVID-19 as well as his hemolytic anemia. However, the following day, the patient’s hemoglobin dropped to 5.2 gm/dL. Thereafter, the heparin infusion was stopped. He received a transfusion of two units of packed red blood cells, and his hemoglobin improved to 7.1 gm/dL. Dexamethasone was switched to methylprednisolone 125 mg every six hours. Rituximab was not given in the setting of COVID-19 infection. The patient’s heparin-induced thrombocytopenia panel came back negative, and the coagulation profile showed prothrombin time (PT) of 14.4 seconds and partial thromboplastin time (PTT) of 34 seconds, 24.95% reticulocyte count, 0.59% absolute reticulocyte count, and 50% ADAMTS13 activity. The patient’s outpatient medications included albuterol, fluticasone-salmeterol, and ipratropium, none of which are known to cause autoimmune hemolytic anemia. His complement and direct antiglobulin test were IgG-positive. Blood cultures were negative.

The patient’s laboratory values confirmed the diagnosis of AIHA. As there was no other apparent cause for the patient’s AIHA, it was attributed to his COVID-19 pneumonia. The patient’s Hgb continued to increase while on steroids, and we started him on apixaban for his bilateral pulmonary emboli. We then discharged the patient on a steroid taper as well as anticoagulation with a follow-up complete blood count (CBC) and an appointment with hematology.

## Discussion

Autoimmune hemolytic anemia (AIHA) is a condition in which the body develops antibodies against red blood cell antigens, activates the immune system, and causes red cell lysis or hemolysis. The first step in the workup is to get a complete blood count (CBC) that shows anemia. The next steps involve obtaining an LDH level, bilirubin, haptoglobin level, reticulocyte count, review of peripheral smear to rule out microangiopathy, and Coombs test. The reticulocyte count will be increased because the bone marrow is increasing the production of new red blood cells, haptoglobin will be low because it is bound to hemoglobin, lactate dehydrogenase will be increased as a result of the cell lysis that occurs, peripheral smear typically shows spherocytosis or microspherocytosis, and a direct antiglobulin test is positive due to the detection of antibodies against RBCs [7]. Our patient had all of the above findings, indicating that he did indeed have hemolytic anemia. Other causes of AIHA, not found in our patient, include autoimmune diseases such as systemic lupus erythematosus (SLE), ABO-incompatible stem cell transplant, hematologic malignancies such as chronic lymphocytic leukemia (CLL) and non-Hodgkin lymphoma, pregnancy, infections, and drugs such as dapsone, penicillin, and its derivatives, or methyldopa. As the patient presented during an episode of COVID-19 pneumonia, we believed that the COVID-19 infection induced AIHA in this case. He was also tested for antinuclear antibody (ANA), and it was negative.

There is a link between COVID-19 and autoimmune hemolytic anemia; however, there is no proven mechanism for these disease processes. A proposed mechanism is that Ankyrin-1 and the spike protein on COVID-19 are molecularly similar, possibly causing the immune system to create antibodies that bind both RBCs and COVID-19. Another proposed mechanism suggests that the SARS-CoV-2-induced cytokine-rich inflammatory response may lead to changes in antigen presentation, generating cryptic antigens [8].

The patient was given dexamethasone 6 mg IV for his COVID-19 pneumonia based on the recommendations in the Randomized Evaluation of Covid-19 Therapy (RECOVERY) Trial [9]. However, it is unclear whether patients with autoimmune hemolytic anemia should be given a higher steroid dosage especially when transfusions are necessary as with respect to our patient. The patient’s hemolytic anemia was worsening, so the dose of steroids was increased to dexamethasone 6 mg IV q8hrs, which was beyond the recommended dose of IV 6 mg/day for symptomatic COVID-19 infection. It is possible that the increased steroids caused a resolution of the AIHA, as the timing would indicate. However, a common course for the treatment of AIHA is to treat the underlying cause, and in this case, that would be COVID-19 infection. It could be that steroids improved our control of COVID-19. This would mean that we just needed to treat the underlying cause of his AIHA but unnecessarily overdosed his steroids, thereby increasing his risk for secondary infections, hyperglycemia, adrenal crisis, and other side effects [10].

## Conclusions

Treatment of autoimmune hemolytic anemia typically involves the administration of glucocorticoids. Increasing steroid dosages inappropriately is especially concerning since an increase in the incidence of side effects from steroids correlates with an increase in steroid dosage. There are currently no guidelines for treating AIHA in the setting of COVID-19 pneumonia, and this case is one that demonstrates the need for further studies to establish such guidelines.

## References

[1] Wang D, Hu B, Hu C (2020). Clinical characteristics of 138 hospitalized patients with 2019 novel coronavirus-infected pneumonia in Wuhan, China. JAMA.

[2] Campos-Cabrera G, Mendez-Garcia E, Mora-Torres M, Campos-Cabrera S, Campos-Cabrera V, Garcia-Rubio G, Campos-Villagomez JL (2021). Autoimmune hemolytic anemia as initial presentation of COVID-19 infection. Blood.

[3] Hindilerden F, Yonal-Hindilerden I, Akar E, Yesilbag Z, Kart-Yasar K (2020). Severe autoimmune hemolytic anemia in COVID-19 İnfection, safely treated with steroids. Mediterr J Hematol Infect Dis.

[4] Jacobs J, Eichbaum Q (2021). COVID-19 associated with severe autoimmune hemolytic anemia. Transfusion.

[5] Jawed M, Hart E, Saeed M (2020). Haemolytic anaemia: a consequence of COVID-19. BMJ Case Rep.

[6] Lazarian G, Quinquenel A, Bellal M (2020). Autoimmune haemolytic anaemia associated with COVID-19 infection. Br J Haematol.

[7] Hill A, Hill QA (2018). Autoimmune hemolytic anemia. Hematology Am Soc Hematol Educ Program.

[8] Al-Kuraishy HM, Al-Gareeb AI, Kaushik A, Kujawska M, Batiha GE (2022). Hemolytic anemia in COVID-19. Ann Hematol.

[9] Horby P, Lim WS, Emberson JR (2021). Dexamethasone in hospitalized patients with Covid-19. N Engl J Med.

[10] Walsh LJ, Wong CA, Oborne J (2001). Adverse effects of oral corticosteroids in relation to dose in patients with lung disease. Thorax.

